# Supplementary material for: Increased Response to Immune Checkpoint Inhibitors with Dietary Methionine Restriction in a Colorectal Cancer Model
Source: Cancers (Basel). 2023 Sep 7;15(18):4467. doi: 10.3390/cancers15184467 (PMC10526448; doi:10.3390/cancers15184467)
Supplement: Supplementary file 1 [file cancers-15-04467-s001.zip › cancers-2585368-supplementary/Table_S1primers.pdf]

		Forward
Human		
	RPLP0	TGT CTG CTC CCA CAA TGA AAC
	HLA-A	CTC TTT GGA GCT GTG ATC ACT
	CD274	CTT TGA GTT TGT ATC TTG GAT GCC
	STING	CTT GAC TGT ATT GTG ACA TGG C
Mouse		
	Rplp0	CGC TTG TAC CCA TTG ATG ATG
	H2Kb	GGT GAC TTT ATC TTC AGG TCT GCT
	Cd274	CCA CAT TTC TCC ACA TCT AGC A

Reverse
TCG TCT TTA AAC CCT GCG TG
GAA GGG CAG GAA CAA MTC TTG
AGG ACT CAC TTG GTA ATT CTG G
GCA TCA AGG ATC GGG TTT ACA G

TTA TAA CCC TGA AGT GCT CGA C
GCT GGT GAA GCA GAG AGA CTC AG
TCC ATC CTG TTG TTC CTC ATT G
