# Supplementary material for: Increased Response to Immune Checkpoint Inhibitors with Dietary Methionine Restriction in a Colorectal Cancer Model
Source: Cancers (Basel). 2023 Sep 7;15(18):4467. doi: 10.3390/cancers15184467 (PMC10526448; doi:10.3390/cancers15184467)
Supplement: Supplementary file 1 [file cancers-15-04467-s001.zip › cancers-2585368-supplementary/Table_S2diets.pdf]

Table S1. Nutritional characteristics of the methionine-adequate (TD.140520) and methionine-restricted (TD.190775) diets used in the study.

Formula	TD.140520 g/Kg	TD.190775 g/kg
Sucrose	445.297	449.28
Corn Starch	198.783	200
Corn Oil	100	100
Cellulose	30	30
Mineral Mix, AIN-76 (170915)	35	35
Calcium Phosphate, dibasic	3	3
L-Alanine	3.5	3.5
L-Arginine HCl	12.1	12.1
L-Asparagine	6	6
L-Aspartic Acid	3.5	3.5
L-Cystine	3.5	3.5
L-Glutamic Acid	40	40
Glycine	23.3	23.3
L-Histidine HCl, monohydrate	4.5	4.5
L-Isoleucine	8.2	8.2
L-Leucine	11.1	11.1
L-Lysine HCl	18	18
L-Methionine	6.5	1.2
L-Phenylalanine	7.5	7.5
L-Proline	3.5	3.5
L-Serine	3.5	3.5
L-Threonine	8.2	8.2
L-Tryptophan	1.8	1.8
L-Tyrosine	5	5
L-Valine	8.2	8.2
Vitamin Mix, Teklad (40060)	10	10
Ethoxyquin, antioxidant	0.02	0.02
% by weight		
Protein	15.3	14.9
CHO	63.3	63.8
Fat	10	10

*- Vitamin Mix, w/o choline, A, D, E; Vitamin E, DL-alpha tocopheryl acetate (500 IU/g; 0.242 g/kg); Vitamin A Palmitate (500,000 IU/g; 0.0396 g/kg); Vitamin D3, cholecalciferol (500,000 IU/g; 0.0044 g/kg).
