# Supplementary figures and images for: Increased Response to Immune Checkpoint Inhibitors with Dietary Methionine Restriction in a Colorectal Cancer Model
Source: Cancers (Basel). 2023 Sep 7;15(18):4467. doi: 10.3390/cancers15184467 (PMC10526448; doi:10.3390/cancers15184467)

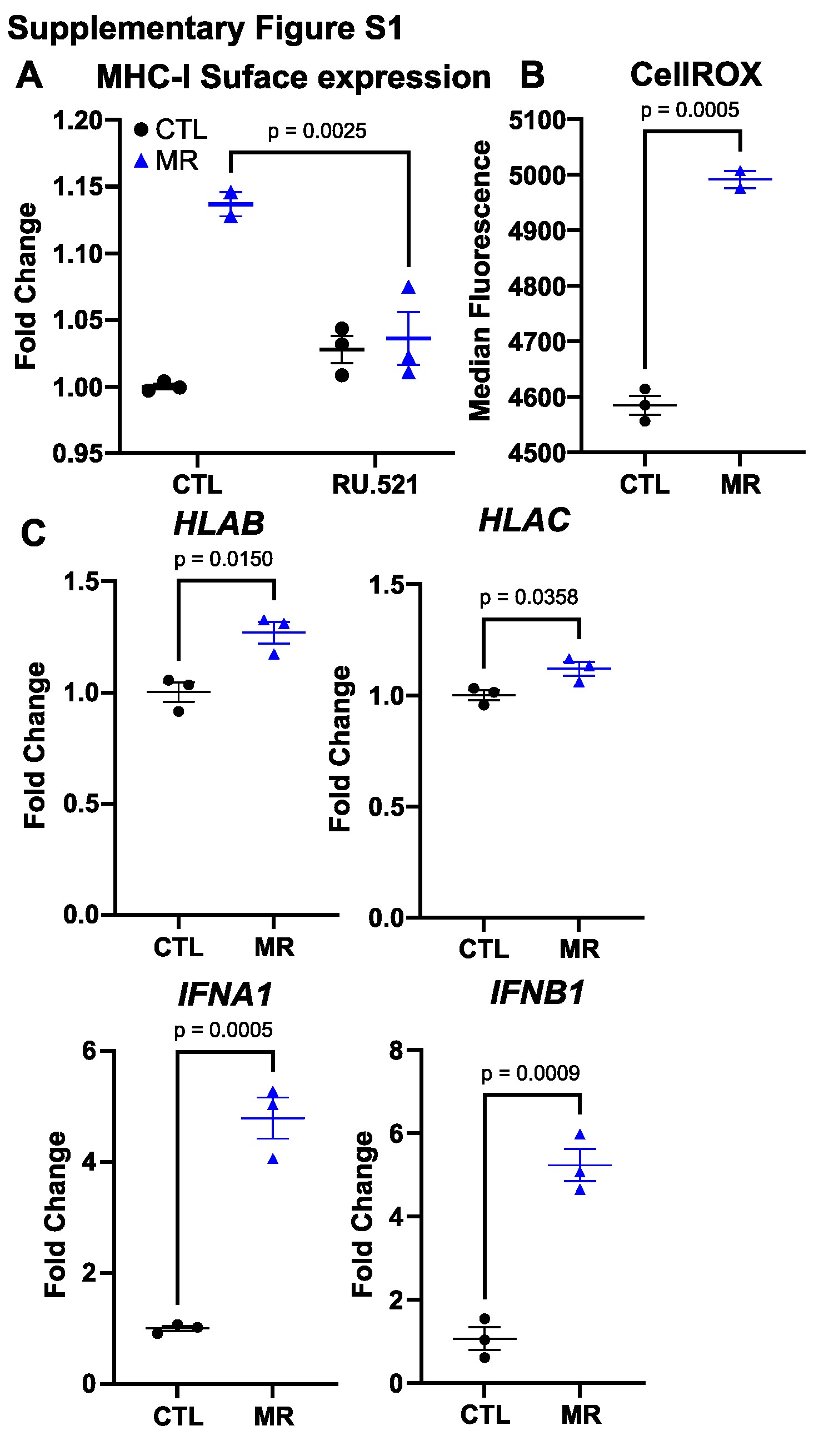

Supplement: Supplementary file 1 [file cancers-15-04467-s001.zip › cancers-2585368-supplementary/SuppFig1_RU.521.jpg]

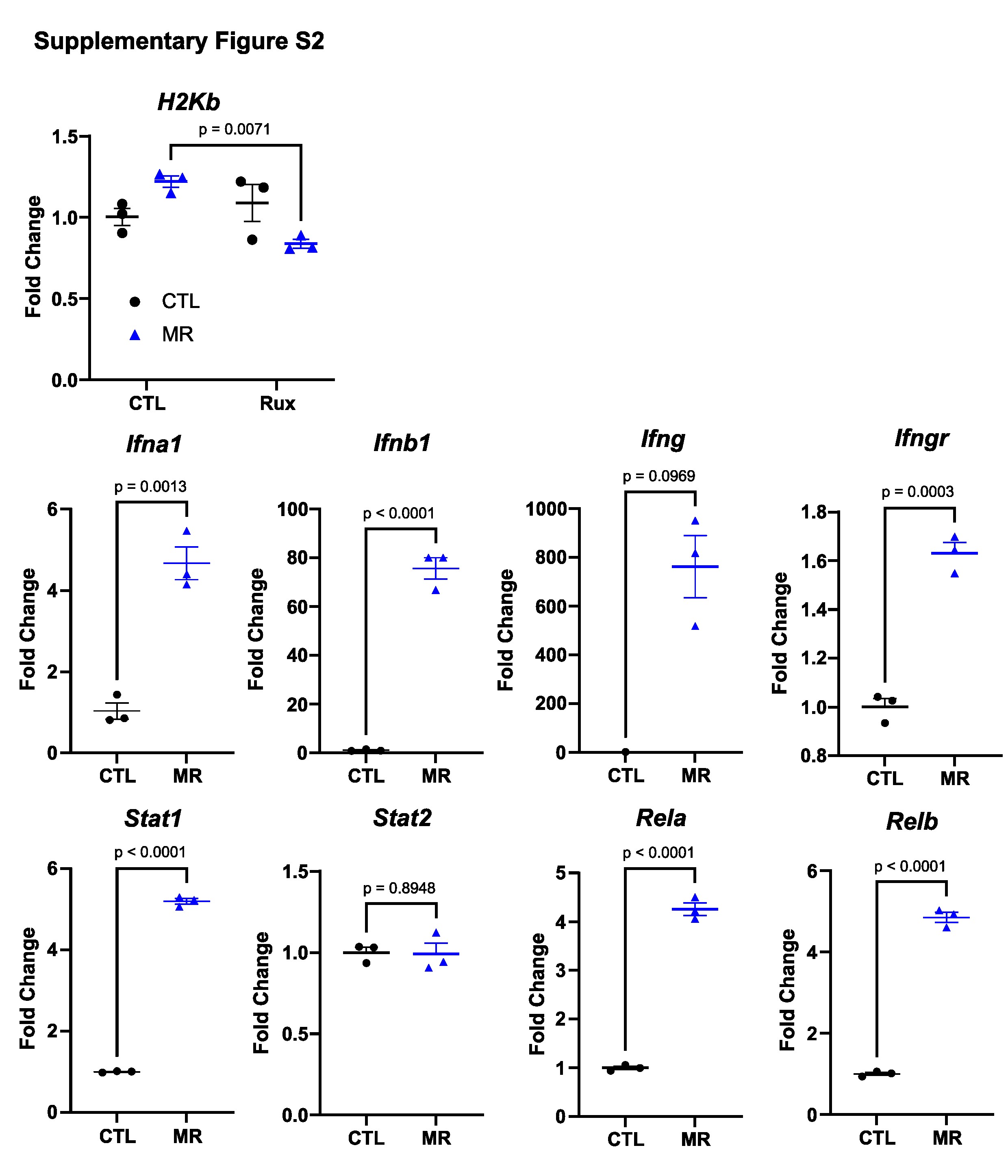

Supplement: Supplementary file 1 [file cancers-15-04467-s001.zip › cancers-2585368-supplementary/SuppFig2_MC38.jpg]

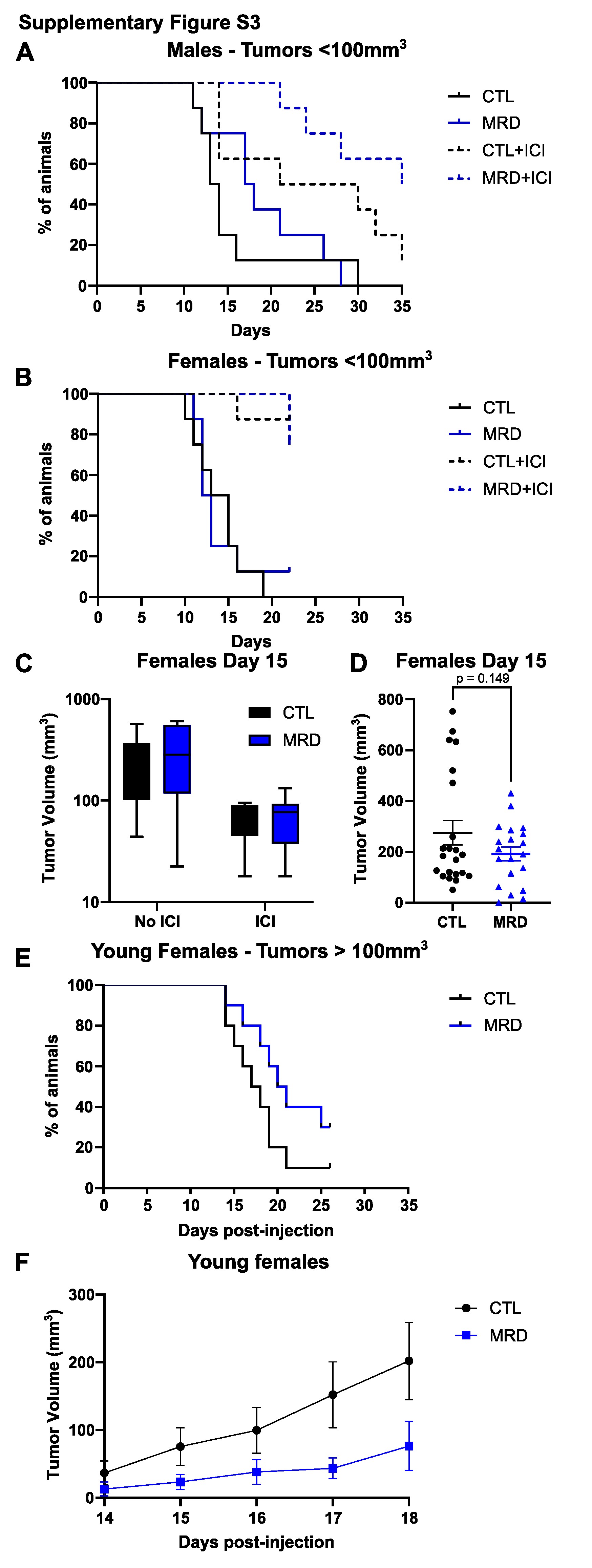

Supplement: Supplementary file 1 [file cancers-15-04467-s001.zip › cancers-2585368-supplementary/SuppFig3_tumors.jpg]

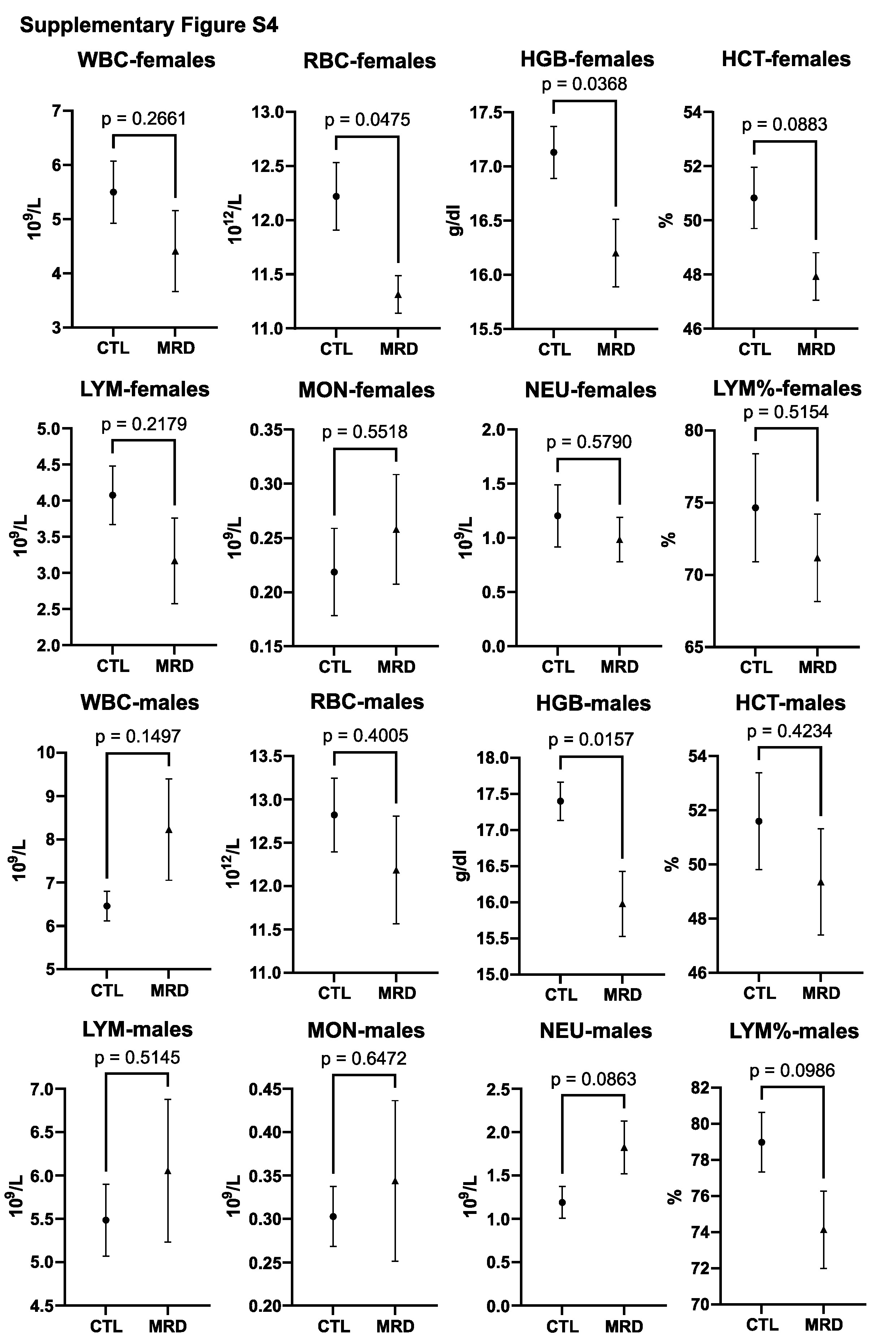

Supplement: Supplementary file 1 [file cancers-15-04467-s001.zip › cancers-2585368-supplementary/SuppFig4_blood.jpg]
